# Case Report: Ivarmacitinib in the treatment of refractory primary cutaneous amyloidosis

**DOI:** 10.3389/fmed.2026.1803727

**Published:** 2026-05-08

**Authors:** Xiaofan Liao, Xian Huang, Guilan Yang, Xia Wang, Qifeng Li, Yuan Lu

**Affiliations:** 1 Department of Dermatology, Affiliated Nanshan Hospital of Shenzhen University, Shenzhen, China; 2 Medical School, Shenzhen University, Shenzhen, China; 3 Department of Dermatology, The Chinese University of Hong Kong, Shenzhen Medical Centre, Shenzhen, China; 4 Department of Pathology, Affiliated Nanshan Hospital of Shenzhen University, Shenzhen, China

**Keywords:** case report, Ivarmacitinib, Janus kinase inhibitor, primary cutaneous amyloidosis, primary localized cutaneous amyloidosis

## Abstract

Primary cutaneous amyloidosis (PCA) is a chronic, severely pruritic dermatosis characterized by amyloid deposition in the dermis. There is currently no standardized treatment for PCA, and its clinical management remains challenging. Ivarmacitinib, a novel, highly selective oral Janus kinase 1 (JAK1) inhibitor, has demonstrated significant efficacy in modulating inflammatory and pruritic pathways in atopic dermatitis (AD), suggesting its potential utility in related conditions. We report a compelling case of a 65-year-old male with a 15-year history of severe, refractory PCA who had failed multiple conventional treatments, including topical corticosteroids, systemic retinoids, and immunomodulators, owing to either poor tolerability or insufficient efficacy. Thus, ivarmacitinib was initiated as a targeted therapeutic intervention, and it resulted in rapid improvement: pruritus subsided within 24 h and resolved completely within one week, followed by significant flattening and fading of hyperpigmented papules over the ensuing weeks, with no adverse events observed during follow-up. This case suggests that ivarmacitinib may be a promising, effective, and well-tolerated therapeutic option for refractory PCA, likely through targeting the JAK/STAT pathway central to disease pathogenesis.

## 1 Introduction

Primary cutaneous amyloidosis (PCA), also known as primary localized cutaneous amyloidosis (PLCA), is a chronic pruritic skin condition characterized by the extracellular deposition of heterogeneous amyloid proteins in the dermis without systemic involvement. PCA manifests in three primary forms: lichen amyloidosis (LA), macular amyloidosis (MA), and nodular amyloidosis (NA). The nodular form can present as a solitary lesion both on the skin (1) and on the mucosal level (2). In the classification of cutaneous forms of amyloidosis, Biphasic amyloidosis (BA), which implies the coexistence of both lichen and macular amyloidosis, has also been described (3). The pathogenesis of PCA remains incompletely understood, though it is believed to involve a combination of environmental and genetic factors. Chronic irritation, itching, and scratching can lead to epidermal damage and keratinocyte degradation, with dermal macrophages and fibroblasts converting keratin peptides into amyloid fibrils that deposit within dermal papillae (4).

The clinical management of PCA poses significant challenges due to its refractory nature to conventional therapies. Current treatment modalities, including topical corticosteroids, oral antihistamines, cyclosporine, retinoic acid, surgery, and CO2 laser therapy, have shown limited efficacy and high recurrence rates (5). Emerging evidence suggests that dysregulated immune responses, particularly those involving Th2 cytokines such as interleukin (IL)-4, IL-13, and IL-31, play a crucial role in the pathogenesis of PCA (6). These cytokines are known to induce pruritus and contribute to skin inflammation and fibrosis.

Ivarmacitinib, a selective JAK1 inhibitor, has demonstrated efficacy in treating multiple inflammatory and immune-related conditions, such as severe atopic dermatitis (AD), by providing rapid relief from pruritus and reducing skin inflammation (7). Given the similarities in the immune dysregulation mechanisms between AD and PCA, JAK1 inhibitors like ivarmacitinib present a promising therapeutic option for PCA.

This article reports a patient with severe cutaneous amyloidosis, who was refractory or intolerant to conventional therapy, following treatment with ivarmacitinib, a selective JAK1 inhibitor. The case highlights the potential of targeting JAK1 as a novel therapeutic strategy for managing PCA, particularly in patients who do not respond to standard treatments.

## 2 Case report

A 65-year-old man was referred to our hospital for a long-standing history of severe itching with brown papules on his limbs and buttocks. The patient first complained of itching on the limbs, buttocks, back and other parts of the body 15 years ago, which often affected sleep, causing difficulty falling asleep or causing awakening due to scratching. Shortly after this itching symptom appeared, “protrusion” appeared on these areas. He was diagnosed with eczema at multiple hospitals and was treated with oral drugs such as levocetirizine, loratadine, and compound glycyrrhizin, along with topical halometasone cream, mometasone furoate cream and tacrolimus cream without clinical improvement. In addition, he was also diagnosed with nodular prurigo and was treated with oral acitretin, along with topical compound clobetasol propionate, calcipotriol and betamethasone, which showed some improvement, with the rash becoming significantly flatter. However, the treatment was discontinued due to the adverse drug reactions such as dry mouth with cracked lips and epistaxis. He stopped the treatment and tried taking thalidomide orally, but experienced numbness in the lips and limbs, so he stopped the medication. The patient had no significant past medical history and no family history of similar skin disorders or other dermatological conditions. He denied any history of atopic dermatitis, allergic rhinitis, asthma, or conjunctivitis.

**Figure 1:**
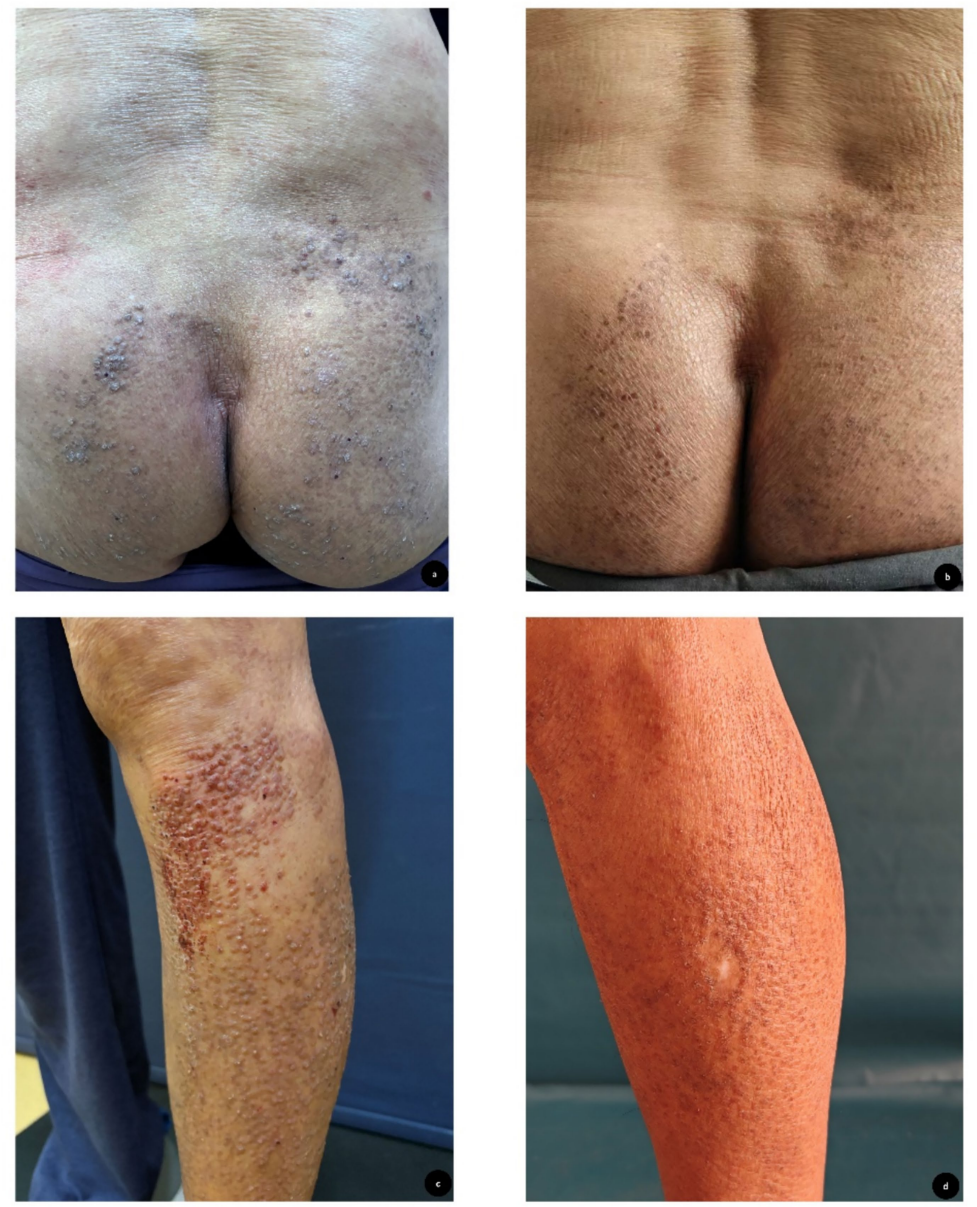
Clinical improvement of skin lesions after two months of ivarmacitinib treatment. **(a,c)** Pretreatment appearance: the skin on the limbs and buttocks is rough and pigmentation. There are numerous dark brown soybean-sized and grain-sized hard papules. Some of the rashes are bleeding and accompanied by excoriation and crust. **(b,d)** The same area marked flattening of papules and significant fading of hyperpigmentation.

**Figure 2:**
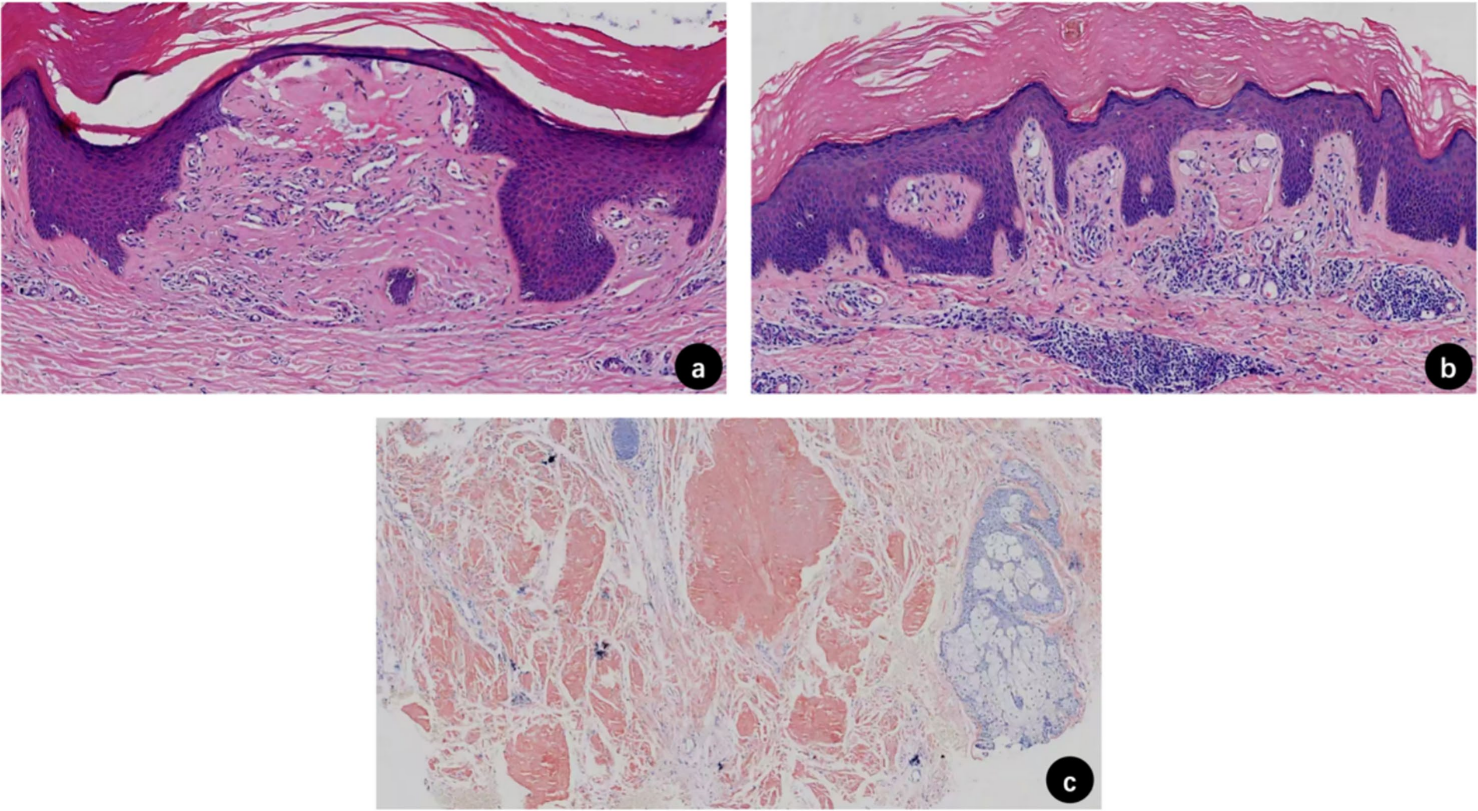
Hematoxylin–Eosin staining **(a,b)**: hyperkeratosis, hypergranulosis, and acanthosis of the epidermis. Within the papillary dermis, there were variably sized, eosinophilic, homogeneous, amorphous deposits. A perivascular inflammatory infiltrate was observed in the dermis, which, composed predominantly of lymphocytes and histiocytes with occasional melanophages. Congo red staining **(c)**: globular and irregular deposits of brick-red material were seen along collagen bundles throughout the dermis.

Physical examination revealed rough and hyperpigmented skin on the limbs and buttocks with numerous dark brown hard papules about 5 mm in diameter. Some of the lesions showed bleeding, excoriation and crust (Figures 1a,c), The patient rated his pruritus as 10 on the Visual Analogue Scale (VAS, 0 = no itch, 10 = worst imaginable itch). A pathological biopsy of the left leg revealed hyperkeratosis and acanthosis of the epidermis. Within the papillary dermis, there were variably sized, eosinophilic, homogeneous, amorphous deposits. A perivascular inflammatory infiltrate was observed in the dermis, composed predominantly of lymphocytes and histiocytes with occasional melanophages (Figures 2a,b). Congo red staining demonstrated globular and irregular deposits of brick-red material along collagen bundles throughout the dermis (Figure 2c). According to the clinical features and these findings, the patient was diagnosed with primary cutaneous amyloidosis.

Given the patient’s documented history of poor tolerance to topical corticosteroids and lack of sustained response to conventional systemic therapies, we chose the ivarmacitinib for this patient. We reviewed the relevant contraindications for medication. Ivarmacitinib, a selective JAK1 inhibitor, was initiated at a standard dose of 4 mg orally once daily, which resulted in alleviation of pruritus rapidly at the first night, as reported by the patient.

Within 1 week, the unbearable pruritus had largely resolved, with the VAS score decreasing dramatically from the baseline score of 10. After 1 month of treatment, the previously hyperkeratotic and lichenified plaques characteristic of lichen amyloidosis (LA) had become notably flattened. Furthermore, the characteristic hyperpigmentation showed significant fading. The VAS score decreased to 0 at this time point. The patient continued ivarmacitinib for two months. By the two-month follow-up assessment, the papules and plaques had largely subsided. The primary residual finding was faint, post-inflammatory hyperpigmentation at the sites of previous lesions (Figures 1b,d).

Throughout the entire two-month treatment period and subsequent follow-up, the patient reported no adverse events and revealed no laboratory abnormalities attributable to ivarmacitinib. The treatment was well-tolerated without interruption.

## 3 Discussion

The management of PCA remains a significant therapeutic challenge, characterized by the persistent lack of a gold-standard treatment. Conventional modalities such as topical corticosteroids, calcineurin inhibitors, systemic retinoids, and immunosuppressants often yield inconsistent results, high relapse rates, and significant adverse effects including cutaneous atrophy, hepatotoxicity, and neurotoxicity. A systematic review by Weidner et al. (4) and an updated appraisal by Hamie et al. (8) both emphasized the urgent need for novel, pathogenesis-targeted therapies. Consequently, patients with severe, refractory disease often remain trapped in a debilitating “itch-scratch” cycle, severely impairing their quality of life.

The pathogenesis of PCA remains unknown but is considered multifactorial, involving genetic predisposition, chronic friction, keratinocyte apoptosis, and dysregulated inflammatory signaling. Recent molecular genetic studies have highlighted the pivotal role of mutations in the oncostatin M receptor *β* (OSMR β) and IL-31RA genes in familial cases (8, 9). These mutations lead to aberrant activation of the downstream JAK/STAT signaling pathway, with the IL-31-mediated pruritus pathway being a central player in disease pathology and symptom burden (10, 11). This understanding has paved the way for exploring JAK inhibitors, which have demonstrated notable efficacy in modulating similar pathways in atopic dermatitis, as promising candidates for refractory PCA (11).

Ivarmacitinib, a highly selective JAK1 inhibitor, strongly and simultaneously blocks the signals of IL-31, IL-4, and IL-13, which orchestrate Th2 inflammation, keratinocyte dysfunction, and fibrosis. This rapidly alleviates itching and breaks the “itch-scratch” cycle, thereby reducing skin immune infiltration and fibrosis (12, 13). Although the medication for PCA was off-label use, successful treatments with the JAK1/2 inhibitor baricitinib and the JAK1 inhibitor abrocitinib have been reported in LA associated with atopic dermatitis (14, 15). Compared with dupilumab which blocks IL-4 and IL-13 signaling only, ivarmacitinib as a JAK1 inhibitor can simultaneously block the signals of IL-31, IL-4, and IL-13, which orchestrate Th2 inflammation. Additionally, ivarmacitinib demonstrates a lower propensity for serious infections and herpes zoster reactivation compared to pan-JAK inhibitors, as supported by clinical trials in atopic dermatitis where infection rates were comparable to placebo.

The differential diagnosis of PCA primarily includes other pruritic dermatoses characterized by papular or lichenified lesions such as lichen simplex chronicus and hypertrophic lichen planus. Lichen simplex chronicus, which results from chronic scratching and typically lacks histologic evidence of amyloid deposition, should be distinguished. Lichen planus, which exhibits violaceous, polygonal papules with Wickham striae, can be distinguished by its histologic features of band-like lymphocytic infiltration and absence of amyloid. Other conditions to be entertained include papular mucinosis, pretibial myxedema, prurigo simplex nodularis, colloid millium, and elephantiasis nostras verrucosa (8). In our patient, the diagnosis of PCA was confirmed by histopathological examination demonstrating characteristic eosinophilic amorphous deposits within the dermal papillae with positive Congo red staining under polarized light, effectively ruling out these differential diagnoses.

This case highlights the feasibility of using ivarmacitinib to treat isolated PCA phenotypes without comorbid AD, expanding the therapeutic landscape for this condition. Compared with existing reports, our findings align with and extend the growing body of evidence supporting JAK/STAT inhibition in PCA. Targeted intervention at the key pathological process—namely, the itching and inflammation mediated by the JAK/STAT pathway—has become a potential new treatment strategy. While this single experience is encouraging, further clinical studies and more cases are warranted to confirm its efficacy, establish optimal dosing, evaluate long-term safety, and elucidate its precise mechanism in PCA.

## Data Availability

The original contributions presented in the study are included in the article/supplementary material, further inquiries can be directed to the corresponding author.
